# The SMAD3 transcription factor binds complex RNA structures with high affinity

**DOI:** 10.1093/nar/gkx846

**Published:** 2017-09-25

**Authors:** Thayne H. Dickey, Anna M. Pyle

**Affiliations:** 1 Department of Molecular, Cellular and Developmental Biology, Yale University, New Haven, CT 06511, USA; 2 Howard Hughes Medical Institute, Chevy Chase, MD 20815, USA; 3 Department of Chemistry, Yale University, New Haven, CT 06511, USA

## Abstract

Several members of the SMAD family of transcription factors have been reported to bind RNA in addition to their canonical double-stranded DNA (dsDNA) ligand. RNA binding by SMAD has the potential to affect numerous cellular functions that involve RNA. However, the affinity and specificity of this RNA binding activity has not been well characterized, which limits the ability to validate and extrapolate functional implications of this activity. Here we perform quantitative binding experiments *in vitro* to determine the ligand requirements for RNA binding by SMAD3. We find that SMAD3 binds poorly to single- and double-stranded RNA, regardless of sequence. However, SMAD3 binds RNA with large internal loops or bulges with high apparent affinity. This apparent affinity matches that for its canonical dsDNA ligand, suggesting a biological role for RNA binding by SMAD3.

## INTRODUCTION

SMAD family transcription factors are central mediators of the TGFβ superfamily signaling pathway (1,2). The first SMAD protein was identified in humans by its frequent mutation in pancreatic cancer, and malfunction of this pathway has since been linked to a variety diseases (3,4). Signaling by TGFβ ligands triggers the phosphorylation, trimerization, and transport of SMAD proteins to the nucleus (5). In the nucleus, SMAD proteins bind to double-stranded DNA (dsDNA) and activate or repress transcription predominantly through the recruitment of histone modification machinery (6,7). Like most transcription factors, SMAD proteins recognize a specific dsDNA sequence known as the SMAD binding element (SBE), which comprises the sequence GTCTG or GTCT (8).

In addition to their canonical role in transcriptional regulation, several SMAD proteins have also been implicated in the processing of primary microRNA (pri-miRNA) transcripts via the recruitment of the microprocessor complex (9). Additional findings suggested a mechanism of action involving the direct interaction of SMAD proteins and pri-miRNA hairpins (10). Furthermore, these interactions were reported to be specific for pri-miRNA hairpins containing the RNA version of the SBE sequence.

The parallels between SMAD RNA and DNA binding are intriguing, but the sequence and structural requirements for RNA binding are poorly defined. The crystal structure of SMAD3 bound to dsDNA revealed a mechanism of sequence recognition typical of transcription factors in which the protein reads a specific pattern of hydrogen bonds in the major groove of the B-form helix (11). The protein makes base-specific contacts via an extended β-hairpin motif, and adjacent DNA-binding domains make no protein-protein contacts, binding without cooperativity. A-form RNA helices are characterized by a narrower and deeper major groove that precludes this mode of recognition. Thus, it is unclear how SMAD might recognize the SBE in the context of a dsRNA hairpin. One possibility is that helical perturbations in the pri-miRNA stem expand the major groove for protein recognition, as seen for other protein/RNA complexes (12–15). However, the catalogue of sequence-specific dsRNA-binding proteins is limited, and usually involves larger helical perturbations than those seen in pri-miRNAs. A better understanding of the sequence and structural features required for RNA binding by SMAD would improve our understanding of miRNA biology, protein-RNA recognition, and the potential for SMAD to interact with other RNA ligands in the cell.

Here, we describe the thermodynamic characterization of SMAD binding to RNA ligands. All work was performed using the SMAD3 protein, which is one of four SMAD proteins previously reported to bind RNA (9). Contrary to previous models, we find no evidence that SMAD3 specifically recognizes the SBE sequence in RNA. In fact, all RNA constructs designed to mimic pri-miRNAs are bound with relatively low affinity, while more complex RNA structures are bound with high apparent affinity. RNAs with large internal loops or junctions bind with apparent affinities that are the same as for dsDNA. Multiple RNA targets effectively compete with SMAD DNA binding, suggesting that SMAD may play an important role in RNA metabolism.

## MATERIALS AND METHODS

### SMAD3 cloning, expression and purification

SMAD3 was expressed and purified as an N-terminally tagged 6xHis-SUMO fusion protein prior to cleavage of the tag for biochemical characterization. A synthetic gene for full-length human SMAD3 was codon optimized for expression in *Escherichia coli* (Integrated DNA Technologies). SMAD3 MH1 (amino acids 1–132) and full-length SMAD3 (1–425) were PCR amplified and cloned into the pET SUMO vector according to manufacturer's recommendations (Thermo Fisher Scientific).

The His-SUMO-SMAD3 fusion protein was expressed in BL21 (DE3) *E. coli*. Cells were transformed with the pET His-SUMO-SMAD3 plasmid and grown at 37°C in luria broth supplemented with 50 μg/ml kanamycin to an OD_600_ of 0.5–0.6. Cells were then cold shocked on ice for 30 minutes with occasional shaking. Protein expression was induced by the addition of 0.5 mM isopropyl β-D thiogalactopyranoside (IPTG) and cells were incubated with shaking for 20 hours at 18°C. Cells were harvested by centrifugation at 5000 RCF for 10 min and resuspended in lysis buffer (20 mM potassium phosphate pH 8.0, 300 mM sodium chloride, 10 mM imidazole pH 8.0, 3 mM βME, 1 EDTA-free protease inhibitor cocktail tablet (Roche), and 5 U/ml benzonase (EMD millipore)).

Cells were lysed by passing through a microfluidizer three times at 15 000 PSI and insoluble material was pelleted by centrifugation at 15 000 RCF for 30 min. Soluble material was mixed with Ni-NTA agarose (Qiagen) preequilibrated in lysis buffer. The slurry was incubated for 1 h with gentle rocking at 4°C and poured into a flex column for purification by gravity flow. The column was washed twice with lysis buffer and once with wash buffer (20 mM potassium phosphate pH 8.0, 300 mM sodium chloride, 25 mM imidazole pH 8.0 and 3 mM βME) before eluting with elution buffer (20 mM potassium phosphate pH 8.0, 300 mM sodium chloride, 100 mM imidazole pH 8.0, 3 mM βME). 6xHis-Ulp1 protease was added to the eluent and dialyzed overnight at 4°C in 6–8 kDa MWCO tubing (Spectrum Labs) in dialysis buffer (50 mM Tris pH 8.0, 150 mM NaCl and 10 mM DTT).

Following cleavage of the His-SUMO tag another Ni-NTA column was used to separate the cleaved His-SUMO tag and His-Ulp1 from SMAD3. The overnight dialysis product was poured over a preequilibrated Ni-NTA column, but SMAD3 was not present in the flow through. SMAD3 was eluted with 20 mM potassium phosphate pH 8.0, 300 mM sodium chloride, 20 mM imidazole pH 8.0 and 3 mM βME. Eluent was concentrated and purified further by gel filtration using a HiLoad 16/600 Superdex 200 column (GE Healthcare) equilibrated in 25 mM Tris–HCl pH 8.0, 115 mM potassium chloride, and 10 mM DTT. Fractions corresponding to highly pure, monomeric SMAD3 MH1 or full-length SMAD3 were pooled, concentrated to ∼100–250 μM, snap frozen in liquid nitrogen and stored at –80°C.

### Oligonucleotide preparation and purification

DNA oligonucleotides were purchased with standard desalting purification (Thermo Fisher Scientific). Short RNA oligonucleotides under 20-nt and construct **12** were synthesized on a MerMade synthesizer (BioAotomation) using standard phosphoramidite chemistry and deprotected as previously described (16).

Long RNA oligonucleotides were transcribed *in vitro* from synthetic ssDNA templates (Thermo Fisher Scientific). Equimolar amounts of ssDNA template and a universal T7 promoter sequence were annealed. One microgram annealed template was then used in a 100 μl transcription reaction containing 5 mM each rNTP, 22 mM magnesium chloride, 40 mM Tris–HCl pH 8.0, 2 mM spermidine, 10 mM DTT, 0.01% Triton X-100, 40 units RNase inhibitor (New England Biolabs) and 5 μl T7 RNA polymerase. Transcription reactions were incubated at 37°C for 2 h.

All RNA was gel purified by denaturing gel electrophoresis. Appropriate bands were excised and RNA was extracted by crush soak in 10 mM MOPS pH 6.0, 300 mM sodium chloride, and 1 mM disodium-EDTA. After extraction, RNA was ethanol precipitated, washed once with 70% ethanol and resuspended in water.

### Oligonucleotide labeling and folding

Oligonucleotides were 5′ end labeled for EMSA experiments. Transcribed RNAs were prepared for end-labeling by dephosphorylation with Antarctic Phosphatase (New England Biolabs) according to manufacturer's recommendations. Following dephosphorylation, enzyme was inactivated by heating to 70°C for 5 min. Oligonucleotides were 5′ end labeled using [ϒ-^32^P] adenosine triphosphate (ATP) and T4 polynucleotide kinase (New England Biolabs) according to manufacturer's recommendations. Free ATP was removed using G25 spin columns (GE healthcare) and oligonucleotides were purified by denaturing gel electrophoresis. Following purification and ethanol precipitation, RNA was resuspended in 25 mM Tris–HCl pH 7.5 and 115 mM potassium chloride and stored at –80°C.

Double-stranded oligonucleotides were created by 5′ end labeling one strand and annealing it with 1.2 molar equivalents of the unlabeled complementary strand. Annealing reactions were performed with at least 170 nM each oligonucleotide in annealing buffer (10 mM Tris–HCl pH 7.5, 50 mM sodium chloride, 1 mM disodium–EDTA). Strands were annealed by heating to 95°C for 5 min and cooled by placing at room temperature.

RNA was folded at low concentrations immediately prior to binding experiments to ensure the absence of dimers or other misfolded conformers. RNA was diluted to less than 10 nM in 25 mM Tris–HCl, 115 mM potassium chloride and 1 mM DTT. The RNA was then placed in a 95°C heating block and allowed to slow cool to room temperature over the course of 60 min.

### Electrophoretic mobility shift assays

For binding reactions, SMAD3 was serially diluted and mixed with trace labeled oligonucleotide at a concentration less than 5 nM. Binding buffer contained 25 mM Tris–HCl, 115 mM potassium chloride, 1 mM magnesium chloride, 1 mM DTT, 20 ng/μl low molecular weight poly I:C (Invivogen), 0.1 mg/ml bovine serum albumin and 10% glycerol. Reactions were incubated on ice for two hours (longer incubation had no affect on results) and loaded onto a 0.75 mm thick native gel made from 6% to 10% acrylamide and 0.5× TBE (44.5 mM Tris–HCl, 44.5 mM boric acid, 1 mM disodium EDTA). Gels were run in 0.5× TBE at 100–120 V for 1 h at 4°C. Gels were dried on whatman paper, exposed to a phosphorimager screen overnight and scanned on a Typhoon FLA 9500 biomolecular imager (GE Healtchare). Data were quantified manually in ImageQuant (GE Healthcare) and corrected for background. Fraction bound was calculated as the total counts from all shifted species divided by the total counts from all bands in a lane. Thus, *K*_D_^app^ reflects the apparent macromolecular dissociation constant of the highest affinity interaction between SMAD3 and the oligonucleotide.

**Figure 1.**
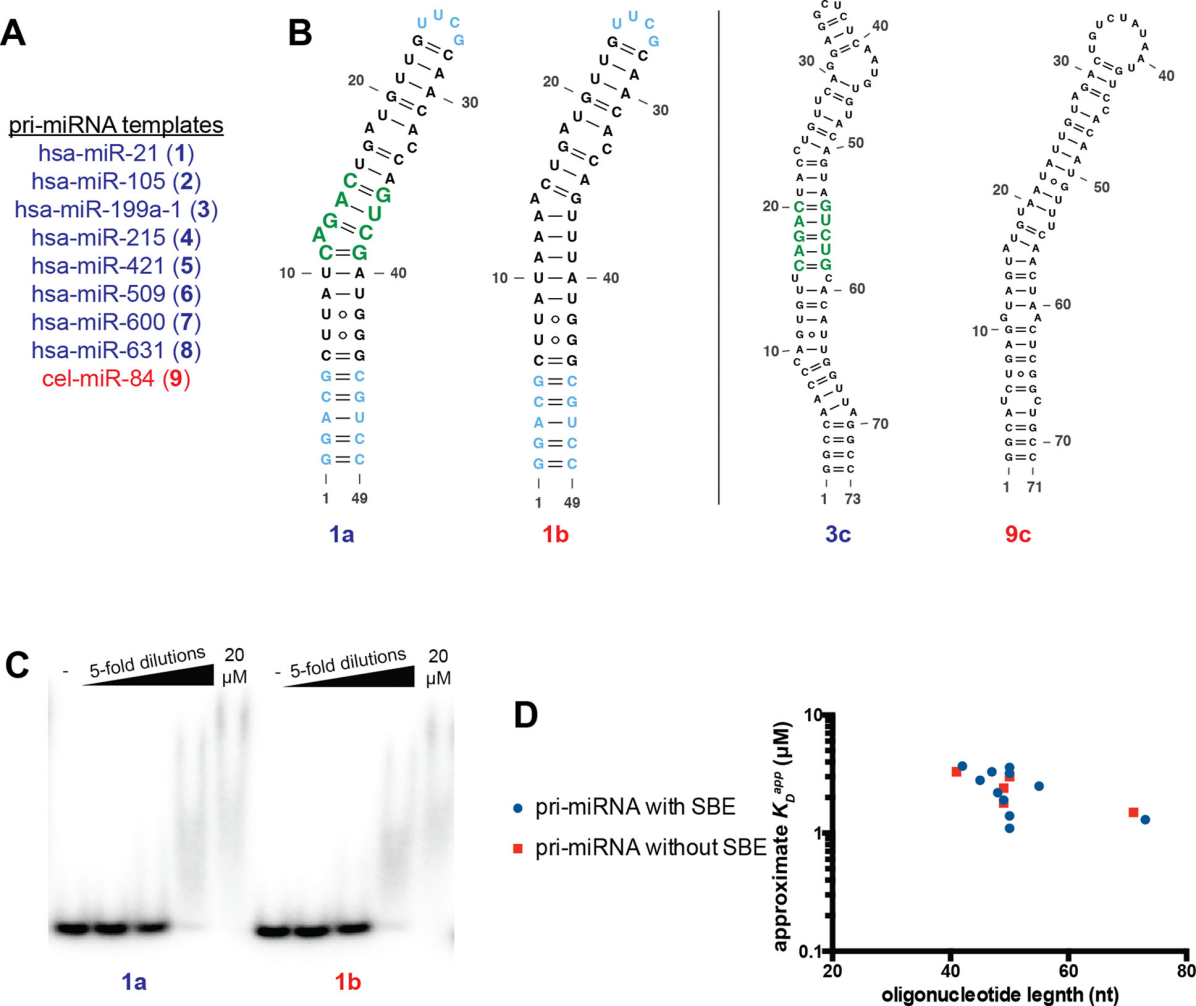
SMAD3 does not specifically bind to SBE-containing pri-miRNAs. (**A**) pri-miRNAs in blue font were selected based on their reported ability to bind SMAD via an SBE motif (10). Pri-miRNAs in red font lack an SBE and are included as negative controls. (**B**) Constructs derived from the pri-miRNAs in panel A were designed to test the effect of the SBE on SMAD binding. Closing basepairs and UUCG tetraloops (cyan) were added to the predicted pri-miRNA secondary structures to stabilize the RNA secondary structure *in vitro*. The local architecture surrounding the SBE (green) was unaltered in the stabilized designs. Negative controls (e.g. **1b**) were created by mutating the SBE sequence. To confirm that our design features did not impact binding, we also tested longer pri-miRNA hairpins without artificial basepairs (right panel). Again, constructs were tested with (**3c**) and without (**9c**) an SBE. (**C**) Binding affinities for each pri-miRNA construct were estimated by incubating SMAD3 MH1 dilutions with each RNA and separating free and bound RNA by EMSA. Representative EMSAs are shown, illustrating that the presence of the SBE does not affect SMAD3 binding. (**D**) Summary of the approximate dissociation constants measured for all pri-miRNA constructs tested. Constructs with putative SBEs are represented by blue circles and those without SBEs are represented by red squares.

Most binding experiments were performed at least in triplicate on different days with SMAD3 concentrations that extended at least 10-fold above and below the *K*_D_^app^. Oligonucleotides that bound weakly, such as **12** and **16**, were measured in duplicate and the initial pri-miRNA binding (Figure 1) was performed as a single screening experiment. Data were fit globally to Equation (1) using GraphPad Prism to calculate *K*_D_^app^ and Hill coefficient.

(1) \begin{equation*}{{Y}} = {{{B}}_{\max }}*{{{X}}^{\rm{h}}}/({K}_{\rm{D}}^{\rm{h}} + {{X}^{\rm{h}}})\end{equation*}

where *Y* is fraction bound, *B*_max_ is the maximal fraction bound, *X* is the concentration of SMAD3, *h* is the Hill coefficient and *K*_D_ is the apparent dissociation constant.

The concentration of SMAD3 in the binding experiments was calculated by absorbance at 280 nm using a theoretical extinction coefficient (26 470 M^−1^ cm^−1^ for SMAD3 MH1 and 68 870 M^−1^ cm^−1^ for FL SMAD 3) (17). Protein specific activity was calculated as ∼70% using an activity titration experiment. This experiment was performed in the same fashion as the binding experiment, but with 2.5 μM dsDNA containing an SBE. The specific activity of SMAD3 was consistent between preps and therefore no correction factor was applied.

### Competition assay

The competition experiment was performed in a similar fashion to the EMSA binding experiments. 1 nM labeled dsDNA containing an SBE was mixed with 350 nM SMAD3 MH1 in binding buffer and incubated on ice for 1 h. Serial dilutions of unlabeled competitor RNA were made and mixed with the preincubated SMAD/dsDNA complex. Competition reactions were incubated on ice for 4 h to reach equilibrium before performing gel electrophoresis, as described for EMSA binding experiments.

At least ten data points were used per experiment and experiments were performed in triplicate with separate dilutions on different days. Competitor concentrations extended at least 10-fold above and below the *K*_D_^app^. Individual replicates were fit to Equation (2) using GraphPad Prism and IC50 values were averaged between replicates. Error bars represent the standard error of the mean of replicate IC_50_ values.

(2) \begin{equation*} {{Y}} = {{{B}}_{\min }} + ({{B}_{\max }} - {{B}_{\min }})/(1 + 10^{({{X}-{\rm log IC}_{50}}))} \end{equation*}

where *Y* is fraction bound, *B*_max_ and *B*_min_ are the maximal and minimum fraction bound, *X* is the concentration of competitor oligo, and IC_50_ is the concentration of competitor oligo that results in a fraction bound halfway between *B*_max_ and *B*_min_.

## RESULTS

### SMAD3 binds RNA without detectable sequence specificity

We designed several RNA constructs to test the model that SMAD3 specifically recognizes the SBE sequence in a pri-miRNA hairpin. Eight pri-miRNAs (**1–8**) were selected as design templates based on their reported interaction with SMAD and the presence of a canonical SBE (Figure 1A) (10). The designed RNA constructs (**1a–8a**) contained the core SBE and five flanking base-pairs from the predicted pri-miRNA secondary structure, so as to maintain local structural elements that might promote binding (Figure 1B left panel and Supplementary Table S1). Five GC-rich closing basepairs and a UUCG tetraloop were added to ensure proper folding *in vitro*. For negative controls, we applied similar design principles to a pri-miRNA lacking the SBE (**9a**) and we mutated the SBE sequences of two designed RNAs (**1b** and **6b**) (Figure 1B left panel and Supplementary Table S1).

**Figure 2.**
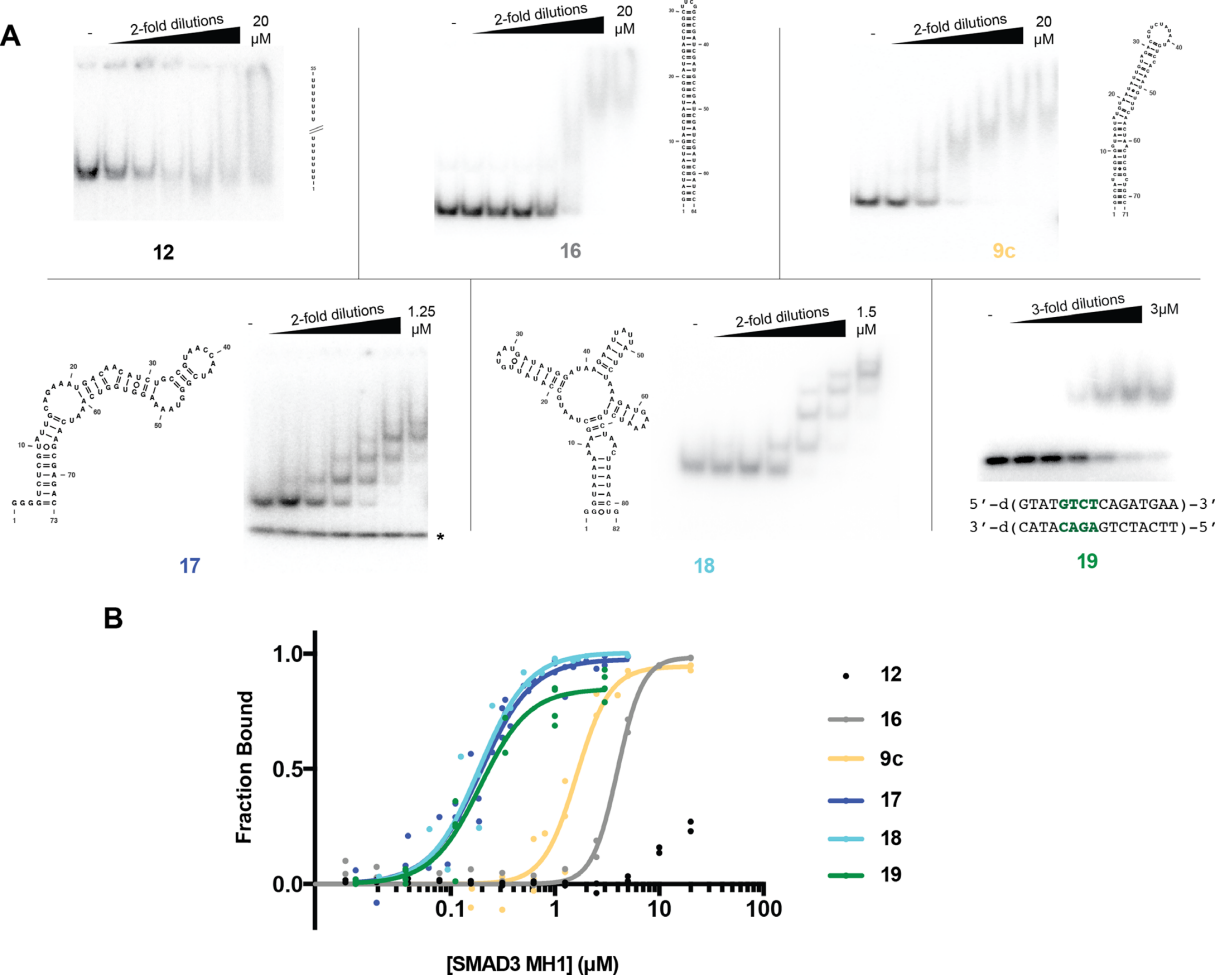
Complex structural features promote SMAD3 binding. (**A**) RNA secondary structures and representative EMSAs designed to test the effect of RNA structure on SMAD3 binding. SMAD3 binds weakly to predominantly single- and double-stranded RNA (**12, 16** and **9c**). However, complex RNA structures (**17** and **18**) are bound with high affinity, comparable to a canonical SMAD3 dsDNA ligand (**19**) containing the SBE sequence (green). Construct **17** misfolds under some conditions and this conformer is marked by an asterisk. (**B**) Data points and global curve fits for SMAD3 RNA and dsDNA binding.

We assessed the sequence specificity of SMAD3 by measuring the approximate dissociation constant for the designed RNAs using an electrophoretic mobility shift assay (EMSA). The DNA-binding domain (MH1) of SMAD3 was used in our binding assays because of its reported ability to confer specificity for the SBE in RNA (10). We verified the activity of the protein and the experimental conditions by recapitulating the well-established affinity for dsDNA containing an SBE (**19**) (Figure 2 and Table 1). Additionally, we confirmed our findings using full-length SMAD3, supporting the conclusion that the MH1 domain confers the complete nucleic acid binding activity of SMAD3 (Supplementary Figure S1).

**Table 1.** RNA and DNA constructs used in quantitative SMAD3 binding experiments

Construct #	RNA/DNA ligand	Length (nt)	Free energy of folding (kcal/mol)^a^	# of large internal loops/junctions^b^ (size)	*K*_D_^app^ (μM)	Hill coefficient
1a	hsa-miR-21 central stem	49	−31.0	0	2.5 ± 0.1	3.1 ± 0.5
1b	hsa-miR-21 central stem ΔSBE	49	−26.9	0	2.7 ± 0.1	3.4 ± 0.4
3c	hsa-miR-199a-1 stem loop	73	−31.4	1 (1 × 4)	1.25 ± 0.05	1.9 ± 0.1
6a	hsa-miR-509 central stem	50	−30.6	0	2.1 ± 0.3	1.8 ± 0.3
6b	hsa-miR-509 central stem ΔSBE	50	−31.4	0	3.0 ± 0.3	3.0 ± 0.7
9a	cel-miR-84 central stem	49	−26.7	0	2.9 ± 0.1	2.7 ± 0.3
9c	cel-miR-84 stem loop	71	−26.9	0	1.6 ± 0.1	2.7 ± 0.4
10	hsa-miR-21 extended	160	−48.3	3 (3 × 3, 2 × 3, 1 × 3)^c^	0.70 ± 0.06	3.2 ± 0.8
11	VAI	155	−85.4	2 (1 × 5, 1 × 2 × 4)	0.11 ± 0.02	1.1 ± 0.1
12	U55	55	N/A	0	>10	N.D.
13	RNA/DNA hybrid duplex with 2 SBEs	16 × 2	−16.4	0	>10	N.D.
14	dsRNA duplex with 2 SBEs	16 × 2	−16.4	0	>10	N.D.
15	miR-21 RNA duplex	16 × 2	−26.1	0	>10	N.D.
16	30-bp hairpin	64	−65.4	0	4.0 ± 0.2	3.6 ± 0.4
17	Group II intron D4A	73	−17.1	2 (1 × 5, 6 × 3)	0.19 ± 0.01	1.7 ± 0.2
17a	D4a truncation 1	56	−14.1	1 (6 × 3)	0.34 ± 0.07	1.7 ± 0.5
17b	D4a truncation 2	44	−15.1	1 (6 × 3)	0.48 ± 0.08	1.8 ± 0.4
17c	D4a truncation 3	35	−7.7	0	1.5 ± 0.3	1.6 ± 0.4
17d	D4a isolated terminal loop	72	−41.9	1 (6 × 3)	0.57 ± 0.02	2.1 ± 0.1
17e	D4a isolated apical loop	72	−45.2	1 (1 × 5)	0.40 ± 0.01	2.2 ± 0.1
18	ai5-gamma D3	82	−17.6	2 (2 × 3, 5 × 3 × 2)	0.19 ± 0.03	1.8 ± 0.4
19	dsDNA with 1 SBE	16 × 2	−16.6	0	0.19 ± 0.02	1.9 ± 0.3
20	NF-κB aptamer	29	−5.1	1 (4 × 3)	4.4 ± 0.6	1.8 ± 0.3
21	RRE	34	−19.5	1 (2 × 3)	3.0 ± 0.3	2.5 ± 0.5

^a^Free energies of RNA folding were calculated with the mfold server using default parameters (50). Duplex energies were calculated using the nearest neighbor method at 1 M NaCl, 37°C and pH 7 (51).

^b^A large internal loop is defined as a region of a stem with unpaired bases on both strands, one of which contains more than one base.

^c^Two of these loops are in the region flanking the central stem loop and are prone to rearrangement in structures predicted by RNA folding algorithms.

The RNA binding experiments revealed weak binding to RNAs with and without the SBE (Figure 1C and D). To confirm that our design features did not interfere with binding, we created several other constructs without artificial closing base-pairs and UUCG tetraloops (**3b** and **4b**) as well as a complete pri-miRNA stem loop sequence (**3c**) (Figure 1B right panel and Supplementary Table S1). We included controls that lack the SBE (**9b** and **9c**), and again, we saw no difference in affinity between RNAs with and without the SBE (Figure 1D). Replicate experiments performed on a subset of these RNAs confirmed these results (Supplementary Figure S2A and Table 1). Thus, SMAD3 binds weakly to pri-miRNA stem loops, regardless of sequence.

### SMAD3 does not preferentially bind pri-miRNAs

Our binding experiments revealed no sequence specificity for SMAD3 binding, but we did see slightly higher affinity for longer RNAs that approached a 1 μM apparent dissociation constant (*K*_D_^app^) (Figure 1D). Indeed, quantitative replicates of several constructs confirm these trends (Table 1 and Supplementary Figure S2A). We further tested the length-dependent binding activity of SMAD3 using a 160-nt construct (**10**), based on hsa-pri-miR-21, which contains the stem loop as well as the predominantly single-stranded flanking regions (Supplementary Figure S2B). We additionally tested a length-matched control RNA (**11**) from adenovirus (VAI) that is largely base-paired, but contains additional structural features that distinguish it from the RNA constructs tested to this point (Supplementary Figure S2C). As expected, the extended construct based on pri-miRNA-21 (**10**) binds with slightly higher affinity than the shorter constructs, demonstrating a length-dependent RNA binding activity (Supplementary Figure S2A). Surprisingly, the length-matched control RNA (**11**) binds 6-fold more tightly than **10**. Thus, SMAD3 can discriminate between length-matched RNAs and does not preferentially bind to pri-miRNAs.

### SMAD3 binds complex RNA structures with high affinity

The ability of SMAD3 to discriminate nearly 10-fold between length-matched RNAs while displaying no sequence specificity suggests that RNA structure plays a role in SMAD3 binding. To further test the role of RNA structure in SMAD3 binding, we measured its affinity for a diverse set of RNA molecules. These RNAs included a single-stranded RNA that contains 55 uracils (**12**), a set of double-stranded RNA and RNA/DNA hybrid duplexes (**13** and **14**), and a 30-basepair stem loop (**16**) (Figure 2A and Supplementary Figure S3). We additionally tested more complex RNA structures that were selected from well-characterized group II intron domains (18,19). The first of these complex RNA structures (**17**) derives from domain four of a group IIC intron and comprises a stem loop with large internal loops (Figure 2A). The second RNA (**18**) derives from domain three of a group IIB intron and comprises a four-way junction with a bulged stem (Figure 2A). SMAD3 binds very poorly to the purely single-stranded and double-stranded RNAs, including those that contain an SBE (**12–15**) (Figure 2 and Supplementary Figure S3). Consistent with this finding, the perfectly base-paired stem loop (**16**) also binds with low affinity (Figure 2). The minor perturbations to base-pairing present in the pri-miRNA stem loops (**1–9**) increase affinity slightly, but the *K*_D_^app^ remains in the micromolar range and no clear shifted species is formed in the EMSA, suggesting the lack of a discrete, stable protein/RNA complex (Figure 2 and Table 1).

While SMAD3 binds poorly to single- and double-stranded RNA, both RNA molecules with more complex structures (**17** and **18**) bind to SMAD3 with significantly higher affinity and they form discrete shifted species that are visible by EMSA (Figure 2). The effect of RNA structure on SMAD3 binding is exemplified by the comparison of **17** and **16**: the complex RNA structure (**17**) binds with a 190 nM *K*_D_^app^ while the perfectly base-paired stem loop (**16**) binds 20-fold weaker, despite being only nine nucleotides shorter (Figure 2B and Table 1). Similarly, **17** binds nearly 10-fold tighter than a pri-miRNA (**9c**) only two nucleotides different in length (Figure 2B and Table 1). Thus, SMAD3 preferentially binds the complex structures present in **17** and **18**.

### Comparison of SMAD3 RNA and dsDNA binding activities

**Figure 3.**
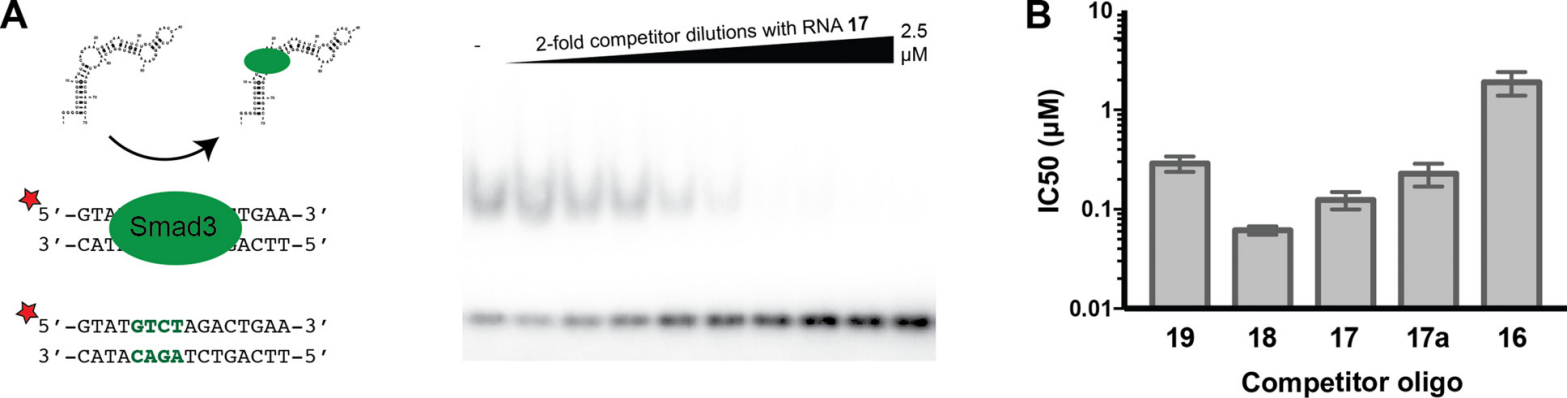
High affinity RNA binding competes with dsDNA binding. (**A)** Representative EMSA competition experiment in which labeled dsDNA (**19**) was preincubated with SMAD3 and unlabeled RNA or DNA was titrated in. (**B)** IC_50_s were calculated for several RNA and DNA oligonucleotides, demonstrating that high-affinity RNAs compete with DNA binding more efficiently than low-affinity RNAs.

The apparent affinity between SMAD3 and these complex RNA structures is comparable to its canonical dsDNA binding affinity. We measured a *K*_D_^app^ of 190 nM between SMAD3 and a dsDNA ligand containing a single SBE (**19**), in agreement with previously reported values (Figure 2) (11). Interestingly, this canonical dsDNA binding affinity is no tighter than that observed for two of the RNA structures (**17** and **18**). To determine if these binding activities are mutually exclusive, we performed competition assays in which unlabeled RNA or DNA was added to a pre-bound SMAD3/dsDNA complex (Figure 3). Indeed, RNA or DNA that binds with high affinity (**17**–**19**) efficiently competes with canonical dsDNA binding at nanomolar concentrations consistent with the *K*_D_^app^, while low-affinity RNAs (**16**) compete much less effectively. Thus, high-affinity RNA binding to SMAD3 competes with canonical dsDNA binding, suggesting that RNA and DNA bind to the same site on SMAD3.

It is important to note that there are differences between the dsDNA and RNA binding activities of SMAD3. dsDNA containing a single SBE (**19**) forms a single shifted species with SMAD3 (Figure 2A, bottom right). Thus, SMAD3 cannot efficiently bind to flanking dsDNA lacking the SBE and the *K*_D_^app^ reflects the microscopic dissociation constant of a single binding event. The complex RNA structures formed by **17** and **18**, however, are capable of binding multiple SMAD3 molecules, as suggested by the multiple shifted species visible by EMSAs (Figure 2A).

### SMAD3 recognizes large internal loops

**Figure 4.**
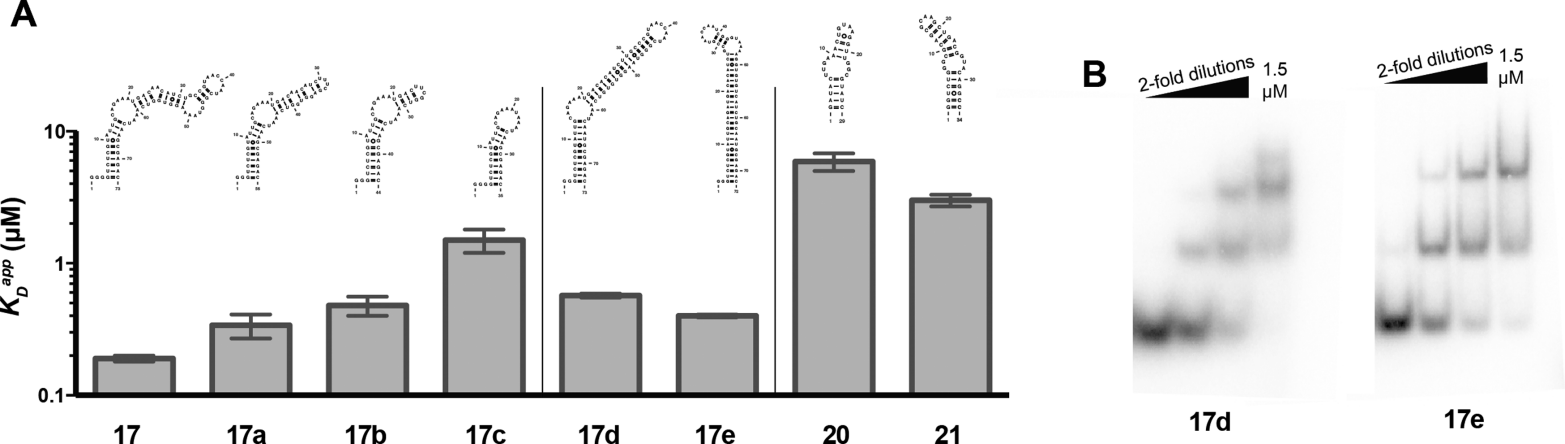
SMAD3 recognizes large internal loops via a mechanism more complex than B-form mimicry. (**A**) Predicted secondary structures and binding affinities of constructs used to determine the minimal structural element recognized by SMAD3. Truncation analysis of **17** (left panel) reveals decreases in affinity upon disruption of each large asymetric internal loop. Either internal loop of **17** is sufficient to confer nanomolar binding in isolation, but not the full affinity conferred by the complete construct **17** (middle panel). The wide-major grooves created by the large internal loops of the NF-κB aptamer (**20**) and RRE (**21**) are insufficient to confer high-affinity SMAD3 binding (right panel). (**B**) Representative EMSAs of SMAD3 binding to constructs containing the isolated internal loops of **17**.

We performed a mutagenesis study on **17** to determine the minimal RNA structural element recognized by SMAD3. First, we performed a truncation experiment in which **17** was progressively shortened and the effect on binding was measured by EMSA (**17a–c**) (Figure 4A). This truncation experiment revealed significant decreases in affinity upon deletion of each internal loop, implicating the internal loops as the sites of SMAD3 binding. To further test this hypothesis, we created full-length versions of **17** in which each internal loop was isolated and the remaining stem was replaced with complementary base-pairs that we previously demonstrated to have low affinity for SMAD3 (**17d** and **17e**). Each of these constructs binds with sub-micromolar apparent affinity, but do not reach the affinity of **17**. Additionally, there are fewer shifted species than with **17**, consistent with a decrease in the number of SMAD3 binding sites (Figure 4B). Together, these findings suggest that SMAD3 binds large internal loops or junctions with moderate affinity and that the presence of multiple binding sites within a single RNA ligand can result in a tight *K*_D_^app^.

### SMAD3 recognizes large internal loops via a mechanism more complex than B-form mimicry

The specificity of SMAD3 for large internal loops and junctions raises the possibility that these RNAs mimic the canonical dsDNA ligand recognized by SMAD3. Large internal loops within RNA stems might be capable of forming distorted helices with expanded major grooves that mimic B-form dsDNA (12,20). We tested this theory using the NF-κB aptamer (**20**) and the Rev-response element from HIV (**21**), both of which contain internal loops that result in helices with expanded major grooves, and which mimic the recognition potential of B-form dsDNA. However, both of these RNAs bind weakly to SMAD3, with affinities that are even lower than **17c**, which is similar length (Figure 4A). These results suggest that SMAD3 recognizes large internal loops and junctions via a mechanism more complex than B-form mimicry.

## DISCUSSION

Here, we describe a novel, high-affinity RNA binding activity for the transcription factor SMAD3. SMAD3 binds RNAs with large internal loops or junctions with mid-nanomolar apparent affinity. This affinity for RNA matches that observed for a specific dsDNA ligand. Therefore, RNA binding has the potential to significantly impact SMAD3 function as well as that of homologous SMAD proteins.

The majority of the human genome is transcribed into RNA, providing a wealth of potential SMAD binding partners (21). Relatively few of these RNAs have been structurally characterized, but those that have suggest the presence of many potential SMAD binding sites. For example, the lncRNA HOTAIR contains 34 internal loops and 19 junctions, the majority of which would be expected to have high affinity for SMAD3 (22). These structures are similarly abundant in other structurally characterized RNAs, suggesting that the transcriptome is filled with potential SMAD3 partners (23–25). However, the stem loops of pri- and pre-miRNAs do not contain such structural motifs, instead forming A-form helices, like that in the crystal structure of a pre-miRNA bound to exportin-5 (26). Other proteins that interact with pri- and pre-miRNAs, such as drosha, DGCR8, dicer and TRBP, contain dsRNA-binding domains (dsRBDs) that canonically bind A-form RNA (27). This suggests a structural requirement for pri-miRNA stem loops to adopt A-form geometry, which is incompatible with SMAD binding. Thus, SMAD3 can bind a wide variety of cellular RNAs, but does not bind single- or double-stranded RNAs, such as the stem loops of miRNA precursors.

The current understanding of when and where SMAD3 exists in the cell suggests multiple ways in which RNA could interact with SMAD3 to regulate TGFβ signaling. Prior to signaling, SMAD3 is localized to the TGFβ receptor at the plasma membrane via direct interaction with the scaffolding protein SARA (28). TGFβ signaling triggers SMAD3 phosphorylation by the TGFβ receptor, promoting SMAD3 trimerization and translocation to the nucleus (5). SMAD3 interaction with SARA and trimerization are mediated by the MH2 domain, leaving the MH1 domain free to interact with RNA (29,30). We do not know if SMAD proteins interact with RNA in the cytoplasm, but such an interaction could serve to transport RNA into the nucleus. The exposed MH1 domain would allow continual interaction with an RNA binding partner, and protein quaternary structure, coupled with RNA tertiary structure, could improve the specificity of SMAD for certain RNAs. Such an RNP would be much smaller than some complexes that utilize the nuclear pore for transport, such as ribosomal subunits, and therefore able to transit the nuclear membrane (31). However, it remains to be seen if importin binding is compatible with RNA binding, and if SMAD does indeed interact with specific RNAs in the cytoplasm.

In the nucleus, SMAD localizes to the chromatin where a large number of nascent and mature RNAs are concentrated (32). These RNAs may influence the genomic localization of SMAD, or *vice versa*, and consequently they may impact the genes that are regulated by TGFβ signaling. Previous experimental results have been interpreted under the assumption that SMAD is a DNA binding protein. However, our results suggest that RNA binding must also be considered. For example, ChIP experiments show SMAD enrichment at enhancer regions, despite the fact that the SBE is not the most enriched sequence motif (33,34). This result could be explained by an interaction between SMAD and chromatin-associated enhancer RNAs. RNA binding may also help explain the pleiotropic nature of SMAD biology. TGFβ signaling has dramatically different effects on different cell types and non-coding RNA is expressed with higher tissue specificity than mRNA (35,36). Thus, cell-specific ncRNAs might differentially regulate SMAD, resulting in different gene expression outcomes for different cell types.

The relatively high affinity and low specificity of SMAD3 for RNA suggests the need for regulation of this binding activity. Many of the most abundant cellular RNAs, such as rRNA, tRNA, 7sk, 7sl, snoRNA, snRNAs, and some mRNAs are present at intracellular concentrations near or above the apparent dissociation constant for SMAD3 (37–39). Therefore, SMAD3 would be predominantly bound to abundant RNAs if it were not for mitigating factors. A higher affinity RNA ligand may exist in the cell and would prevent nonspecific interactions with abundant structured RNAs. Protein complexes or compartmentalization may also help obscure potential SMAD binding sites on abundant RNAs. However, pathologies or experiments that lead to an increase in RNA concentration without a concomitant increase in protein partners would lead to the exposure of many potential SMAD binding sites. Thus, RNA overexpression could lead to aberrant interactions with SMAD.

Our results have additional implications regarding the requirements for SMAD DNA binding. We have shown that multiple RNA binding sites achieve the same effective dissociation constant and compete with specific DNA binding. When localized to the chromatin, SMAD will encounter a large number of RNA binding sites (32). Therefore, stable DNA binding requires a significantly higher affinity for DNA than previously appreciated. Interactions between SMAD and other DNA-binding transcription factors may cooperatively increase the affinity of the complex, and larger enhanceosome complexes may be required to specifically target SMAD to the proper genomic location (40,41). RNA and DNA may also work synergistically to localize SMAD to a specific genomic location. While RNA and DNA binding are competitive for a single SMAD monomer, the formation of a SMAD trimer, or a larger protein complex, would allow concurrent binding of DNA and RNA. The formation of such a complex would improve specificity and affinity, potentially helping localize SMAD to a specific location of the chromatin.

The extrapolation of our findings to other transcription factors is still uncertain, but our results agree with the few transcription factors that have been well characterized. Several transcription factors have been suggested to bind RNA, but very few have been quantitatively characterized (42). Our results demonstrate the need to confirm these interactions *in vitro* as indirect effects complicate cell-based experiments. The handful of transcription factors that have been characterized agree well with our observations on SMAD3. TFIIIA, NF-κB, RUNX1, GR, and now SMAD3 each bind RNA with a mid- to low-nanomolar apparent *K*_D_ within an order of magnitude of their *K*_D_ for DNA (43–47). TFIIIA uses different domains to bind RNA and DNA, but the other transcription factors are capable of binding both RNA and DNA via overlapping interfaces (15,20,48,49). Interestingly, each of the RNA binding partners also contains at least one large internal loop or junction. Further experiments are needed to understand the themes in transcription factor/RNA interaction, but our results suggest a potentially important biological role for RNA binding by SMAD, and perhaps other transcription factors.

The RNA binding ability of SMAD must be considered in the design and interpretation of future experiments. We have demonstrated a high-affinity interaction with RNA that is not sequence specific, as previously described, but instead has moderate specificity for internal loops or junctions. The magnitude of this interaction suggests a novel role for RNA in TGFβ signaling.

## Supplementary Material

Supplementary Data
