# Supplementary material for: The SMAD3 transcription factor binds complex RNA structures with high affinity
Source: Nucleic Acids Res. 2017 Sep 25;45(20):11980–8. doi: 10.1093/nar/gkx846 (PMC5714123; doi:10.1093/nar/gkx846)
Supplement: Supplementary Data [file gkx846_supp.pdf]

Figure S1

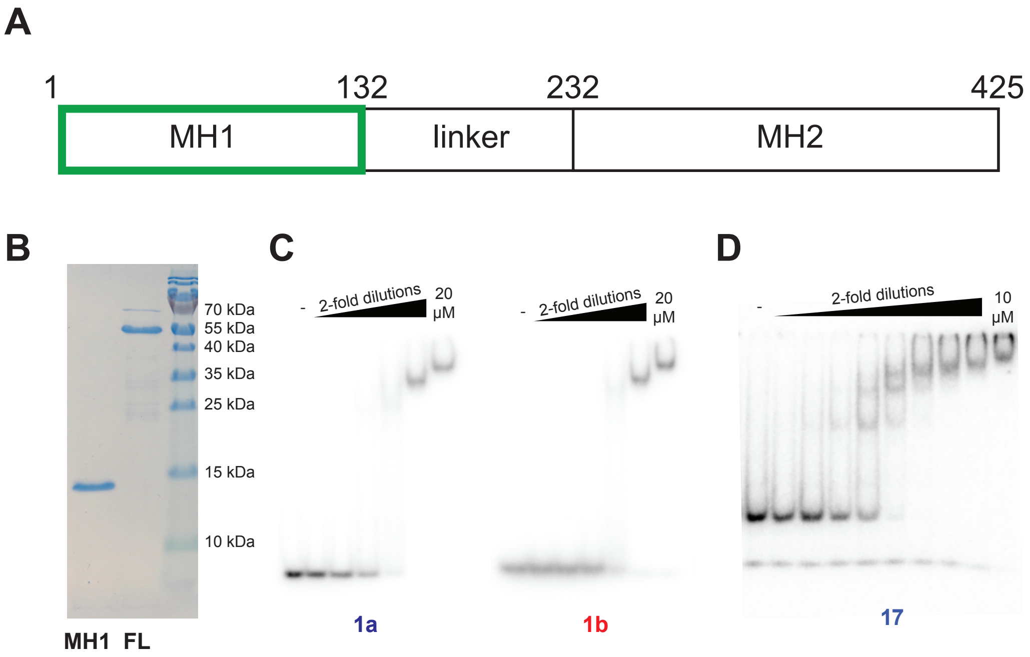
A
1
132
232
425
MH1
linker
MH2
B
70 kDa
55 kDa
40 kDa
35 kDa
25 kDa
15 kDa
10 kDa
MH1 FL
C
- 2-fold dilutions 20 µM
- 2-fold dilutions 20 µM
1a
1b
D
- 2-fold dilutions 10 µM
17

Figure S2

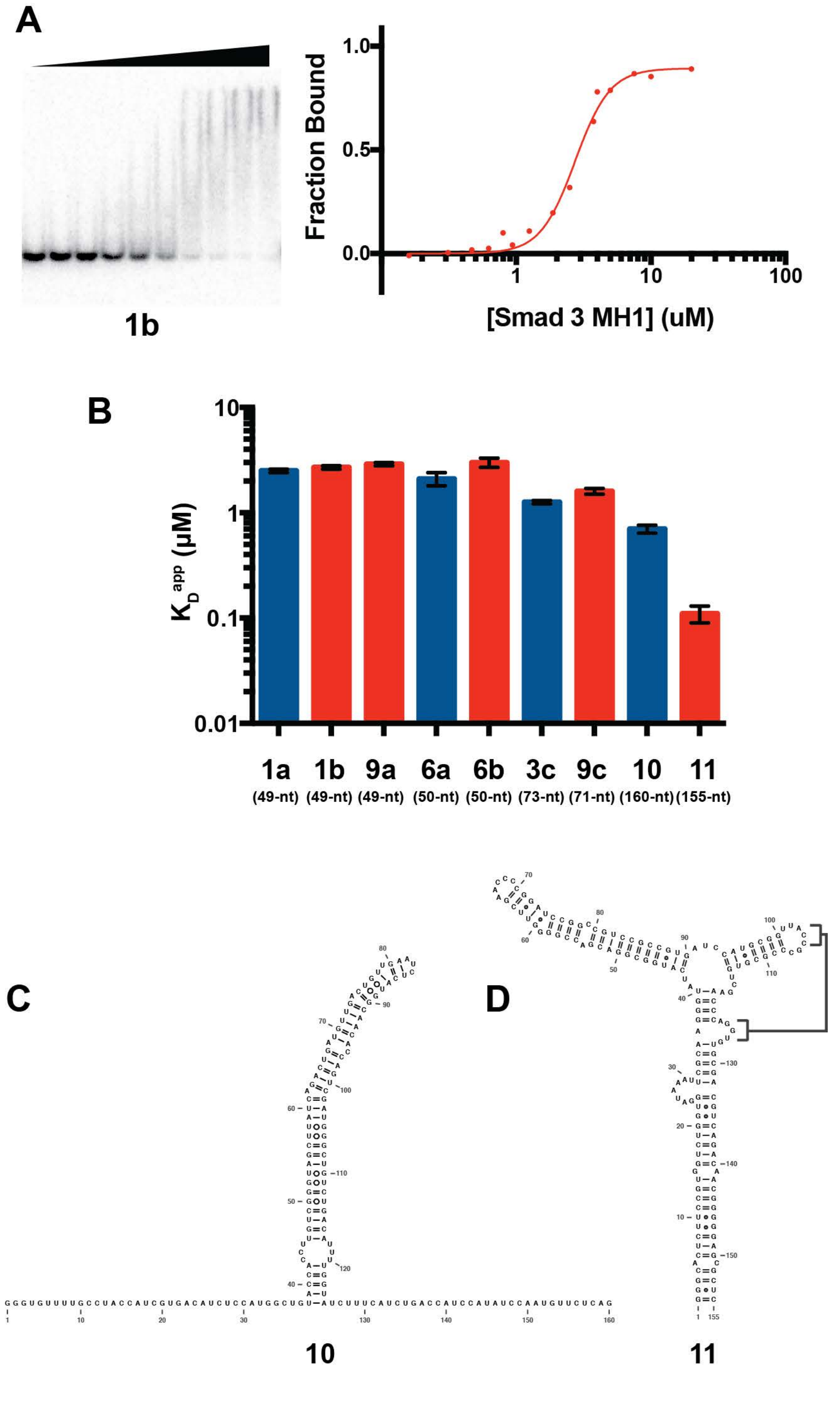

A
1b
Fraction Bound
1.0
0.5
0.0
1
10
100
[Smad 3 MH1] (uM)
B
K_D^{app} (μM)
10
1
0.1
0.01
1a (49-nt)
1b (49-nt)
9a (49-nt)
6a (50-nt)
6b (50-nt)
3c (73-nt)
9c (71-nt)
10 (160-nt)
11 (155-nt)
C
D
10
11

Figure S3

A

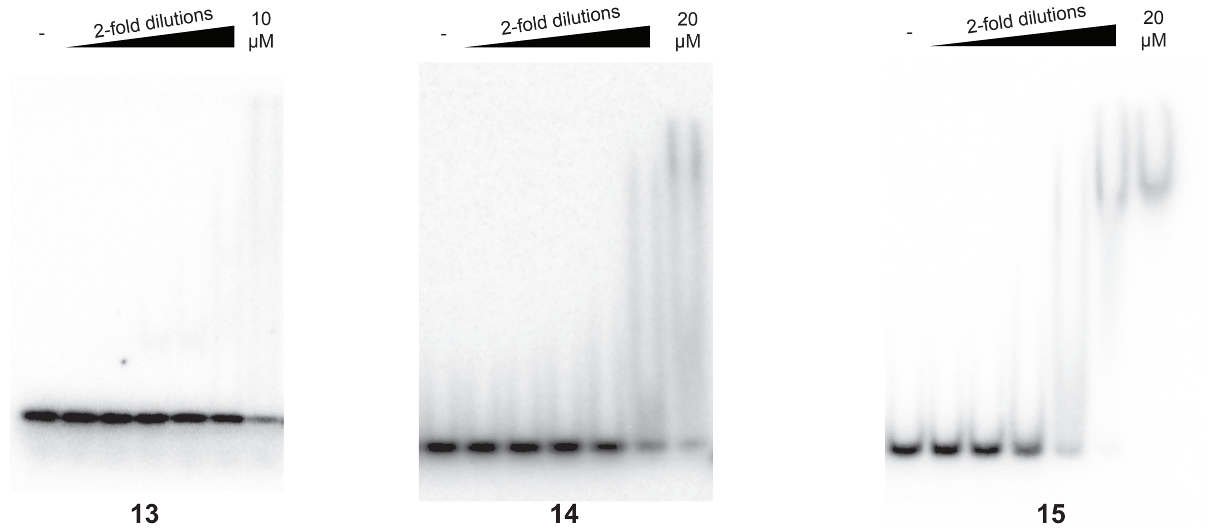

**13**	**14**	**15**
5′-r(GUAU**GUCUAGAC**UGAA)-3′	5′-r(GUAU**GUCUAGAC**UGAA)-3′	5′-r(UCAACAUCA**GUCUG**AUAAGCUA)-3′
3′-d(CATA**CAGATCTG**ACTT)-5′	3′-r(CAUA**CAGAUCUG**ACUU)-5′	3′-r(AGUUGUAGU**CAGAC**UAUUCGAU)-5′

Supplementary Table 1. Complete list of RNA sequences used in this study

Construct number	Template pri-miRNA	Sequence
**1a**	hsa-miR-21 central stem	GGACGCUUAUCAGACUGAUGUUGUUCGCAACACCAGUCGAUGGGCGUCC
**1b**	hsa-miR-21 central stem ΔSBE	GGACGCUUAUAAAACUGAUGUUGUUCGCAACACCAGUUUAUGGGCGUCC
**2a**	hsa-miR-105 central stem	GGACGAUGCUCAGACUCCUGUGUUCGCACGGAUGUUUGAGCAUCGUCC
**3a**	hsa-miR-199a1 central stem	GGACAACCAGUGUUCAGACUACCUGUUUCGACAGUAGUCUGCACACUGGUUGUCC
**3b**	hsa-miR-199a1 minimal SBE	GGACAAUCAGACUACCUGUUUCGACAGGUAGUCUGCUUGUCC
**3c**	hsa-miR-199a1 stem loop	GGCCAACCCAGUGUUCAGACUACCUGUUCAGGAGGCUCUCAAUGUGUACAGUAGUCUGCACAUUGGUUAGGCC
**4a**	hsa-miR-215 central stem	GGACGAUUGACAGACAAUAUGCGUUCGCGCAGUUUGUCUGUCAUUCGUCC
**4b**	hsa-miR-215 natural loop	GGACGAUUGACAGACAAUAUAGCUGAGUUUGUCUGUCAUUCGUCC
**5a**	hsa-miR-421 central stem	GGACGUUAAAUGUUUGUUGAGCGUUCGCGCUCAACAGACAUUAAUCGUCC
**6a**	hsa-miR-509 central stem	GGACGUACUGCAGACAGUGGGCGUUCGCGCGUACGUCUGUGGGUACGUCC
**6b**	hsa-miR-509 central stem ΔSBE	GGACGUACUGCCAGAAGUGGGCGUUCGCGCGUACUCUGGUGGGUACGUCC
**7a**	hsa-miR-600 natural loop	GGACGCUCUUGUCUGUCAGGCAGUGGAGUUACUUACAGACAAGAGCGUCC
**8a**	hsa-miR-631 central stem	GGACGUGGCCCAGACCUCAGCUUCGGCUGAUGGACUGAGUCACGUCC
**9a**	cel-miR-84 central stem	GGACGUGAGGUAGUAUGUAAGCGUUCGCGCUUUCAACUAACUCGCGUCC
**9b**	cel-miR-84 short	GGUGAGGUAGUAUGUAAGCUUCGGCUUUCAACUAACUCGCC
**9c**	cel-miR-84 stem loop	GGCAUCUGAGGUAGUAUGUAAUAUUGUAGACUGUCUAUAAUGUCCACAAUGUUUCAACUAACUCGGCUGCC
**10**	hsa-miR-21 extended	GGGUGUUUUGCCUACCAUCGUGACAUCUCCAUGGCUGUACCACCUUGUCGGGUAGCUUAUCAGACUGAUGUUGACUGUUGAAUCUCAUGGCAACACCAGUCGAUGGGCUGUCUGACAUUUUGGUAUCUUUCAUCUGACCAUCCAUAUCCAAUGUUCUCAG
**11**	VAI	GGGCACUCUUCCGUGGUCUGGUGGAUAAAUUCGCAAGGGUAUCAUGGCGGACGACCGGGGUUCGAACCCCGGAUCCGGCCGUCCGCCGUGAUCCAUGCGGUUACCGCCCGCGUGUCGAACCCAGGUGUGCGACGUCAGACAACGGGGGAGCGCUC

**12**	U55	UUUUUUUUUUUUUUUUUUUUUUUUUUUUUUUUUUUUUUU UUUUUUUUUUUUUUUU
**13**	RNA/DNA hybrid duplex with 2 SBEs	5’ – r(GUAUGUCUAGACUGAA) – 3’<br>3’ – d(CATACAGATCTGACTT) – 5’
**14**	dsRNA duplex with 2 SBEs	5’ – d(GTATGTCTAGACTGAA) – 3’<br>3’ – d(CATACAGATCTGACTT) – 5’
**15**	miR-21 RNA duplex	5’ – r(UCAACAUCAGUCUGAUAAGCUA) – 3’<br>3’ – r(AGUUGUAGUCAGACUAUUCGAU) – 5’
**16**	30-bp hairpin	GGAUCGAUCGAUCGAUCGGCAUCGAUCGGCUUCGGCCGAUCG AUGCCGAUCGAUCGAUCGAUCC
**17**	Group II intron D4A	GGGGUCUCGUAUUGCAGAAAUGACAACAUCUGCCGUAACCAA UCGGGUAAAAGGUGGUCAAAUCAAGCGAGAC
**17a**	D4a truncation 1	GGGUCUCGUAUUGCAGAAAUGACAACAUCUUUCGAGGUGGUC AAAUCAAGCGAGAC
**17b**	D4a truncation 2	GGGUCUCGUAUUGCAGAAAUGACUUCGGUCAAAUCAAGAGAG AC
**17c**	D4a truncation 3	GGGUCUCGUAUUGCAGAAAUAAAUCAAGCGAGAC
**17d**	D4a isolated terminal loop	GGGUCUCGUAUUGCAGAAAUGACACAUCUUGCCCGUAACCAA UCGGGUAAGGUGUGUCAAAUCAAUGCGAGAC
**17e**	D4a isolated apical loop	GGGUCUCGUAUUGCAGAUGACACAUCUGCCGUAACCAAUCGG GUAAAAGGUGUGUCAUCUGCAAUGCGAGAC
**18**	ai5-gamma D3	GGGUAUAAAAAGCUAAUGCCAUAUUGUAAUGAUAUGGAUAA GAAUUAUUAUUCUAAAGAUGAAAAUCUGCUAACUUAUACUG
**19**	dsDNA with 1 SBE	5’ – GTATGTCTCAGATGAA – 3’<br>3’ – CATACAGAGTCTACTT – 5’
**20**	NF-KB aptamer	GAUACUUGAAACUGUAAGGUUGGCGUAUC
**21**	RRE	GGUCUGGGCGCAGCGCAAGCUGACGGUACAGGCC

Supplementary Figure 1
SMAD3 RNA binding is conferred by the MH1 domain. A) SMAD3 domain map with the nucleic acid-binding MH1 domain highlighted in green. **B)** SDS-PAGE gel of the purified protein constructs used for all experiments. **C)** Representative EMSAs of full-length SMAD3 binding to pri-miRNA constructs with and without the SBE. This binding recapitulates the affinity and the lack of sequence specificity conferred by SMAD3 MH1. **D)** Representative EMSA of full-length SMAD3 binding to **17**. This binding recapitulates the high affinity for complex RNA structures conferred by SMAD3 MH1.

Supplementary Figure 2
SMAD3 does not preferentially bind pri-miRNA stem loops. A) Representative EMSAs and curve fits for replicate experiments performed on pri-miRNA constructs **B)** K_D^{app} values calculated from replicate experiments on pri-miRNA constructs. pri-miRNA constructs containing putative SBEs are shown in blue and negative controls are shown in red. **C)** Secondary structure of the miRNA construct **10** containing the central stem loop and flanking single-stranded regions. RNA folding algorithms predict a stable central stem and many unstable conformations for the flanking regions. **D)** Secondary structure of the length matched control RNA construct **11.** The secondary structure is based on experimental probing data (51).

Supplementary Figure 3
SMAD3 does not bind dsRNA or RNA/DNA hybrid duplexes. Representative EMSAs of poor SMAD3 binding to RNA-containing duplexes.

Supplementary Table 1
Complete list of RNA sequences used in this study
